# Paediatric refraction practices: access to cyclopegia, barriers, and disparities among eye care professionals in Kuala Lumpur

**DOI:** 10.1186/s12887-026-07069-x

**Published:** 2026-06-05

**Authors:** Effendy Bin Hashim, Hussein Waheeda-Azwa, Azlina Mokhtar, Priya Morjaria

**Affiliations:** 1 Department of Eye and Vision Sciences, Institute of Life Course and Medical Sciences, University of Liverpool, William Henry Duncan Building, West Derby Street, Liverpool, L7 8TX UK; 2 Ministry of Health Malaysia, Training Division, Federal Government Administrative Centre, Level 6, No. 26, Persiaran Perdana Putra, Precint 3, Putrajaya, 62675 Malaysia; 3 Department of Ophthalmology, School of Medical Sciences, Universiti Sains Malaysia, Kelantan, Kubang Kerian Malaysia; 4 Faculty of Medicine and Health Sciences, Universiti Sains Islam Malaysia, Persiaran Ilmu, Persiaran Ilmu, Bandar Baru Nilai, Negeri Sembilan 71800 Malaysia; 5 International Centre for Eye Health, Faculty of Infectious & Tropical Diseases, London School of Hygiene and Tropical Medicine, Keppel Street, London , WC1E7HT UK

**Keywords:** Accommodative esotropia, Amblyopia, Cycloplegia, Eye care professionals, Latent hyperopia, Myopia, Paediatric refraction, Ophthalmologist, Optician, Optometrist

## Abstract

**Background:**

Accurate paediatric refraction is essential for detecting and managing vision issues, including latent hyperopia, amblyopia, accommodative esotropia, and progressive myopia. Cycloplegic agents are critical for obtaining precise measurements in children, yet their use varies due to professional, regulatory, and systemic barriers. In Malaysia, where refractive services are divided between the public and private sectors, limited data exist on how eye care professionals implement cycloplegic refraction in paediatric practice.

**Methods:**

This cross-sectional study employed a self-administered questionnaire to survey licensed ophthalmologists, optometrists, and opticians within a 50-km radius of Kuala Lumpur. Participants were recruited through electronic invitations and purposive field visits. Descriptive statistics summarised paediatric refraction practices by profession and sector, and exploratory chi-square, Fisher’s Exact, and trend analyses assessed sectoral differences.

**Results:**

Among 222 respondents (61.3% private; 38.7% public), analysis revealed a statistically significant sectoral divide in practice patterns (*p* < 0.001). Public-sector optometrists administered cycloplegia to 82.3% of children aged 5–9 years, compared with 15.7% in private practice, representing an Odds Ratio of 24.97 (95% CI 10.35–60.21; χ² = 63.84,* df = 1*, *p* < 0.001). Private opticians reported 0% cycloplegia use across all age groups, while 79.5% of private optometrists who never used cycloplegia cited ‘*prohibited by law*.’ Public ophthalmologists commonly delegated refraction, with only 8.3% performing refraction in children aged 5–9 years. Free-text responses highlighted additional barriers, including workflow constraints, limited training, and misconceptions about the role of cycloplegia.

**Conclusions:**

Paediatric refraction practices in Kuala Lumpur vary substantially across sectors and professional groups. Despite its clinical importance, cycloplegia remains under-utilised—particularly in private practice. These exploratory findings highlight the need to improve access to cycloplegic agents, clarify professional scope, and address legal and institutional barriers as priorities for strengthening paediatric eye care in Malaysia.

**Supplementary Information:**

The online version contains supplementary material available at 10.1186/s12887-026-07069-x.

## Introduction

Accurate assessment of refractive errors in children is crucial for supporting normal visual development and preventing conditions like amblyopia and fully accommodative esotropia. However, obtaining precise measurements can be difficult due to strong accommodation in younger children. Cycloplegic agents are widely recommended in paediatric eye care to temporarily paralyse the ciliary muscle, thereby preventing accommodation and enabling more accurate measurement of refractive status [1]. Without cycloplegia, children risk receiving incorrect prescriptions or misdiagnoses, particularly with latent hyperopia or other accommodative issues. The World Health Organization (WHO) recommends using cycloplegic refraction in children to ensure proper correction and minimise the risk of progressive myopia [2].

Cycloplegia aids in myopia management and helps detect hyperopia, latent hyperopia, amblyopia, and accommodative strabismus, which may not be apparent during non-cycloplegic refraction. Cycloplegic refraction is particularly crucial during initial evaluations and for younger children, where accommodation is at its strongest and most variable [3].

Myopia remains a significant public health concern, linked to complications such as retinal detachment, glaucoma, and myopic maculopathy [4]. Cycloplegic refraction is essential for both myopia management and the accurate diagnosis of paediatric refractive conditions. Early detection and appropriate correction are vital for preventing long-term visual impairment and supporting optimal visual development. Cycloplegic refraction thus remains a cornerstone of comprehensive paediatric eye care.

In paediatric refraction practice, the use of cycloplegic agents varies among eye care professionals depending on their training, experience, patient demographics, and institutional protocols [5]. Some follow established guidelines based on age or refractive findings, while others adopt more personalised approaches [6]. Regulatory restrictions in specific settings—particularly in the private sector—may further restrict the use of cycloplegia in children.

In Malaysia, cyclopentolate and tropicamide are classified as controlled substances under the Poisons Act 1952 (Act 366), meaning they may only be supplied or administered by registered medical practitioners, dentists, or veterinary surgeons [7]. Although the Optical Act 1991 permits optometrists to use diagnostic agents for refractive purposes, it does not confer independent authority to administer or prescribe these agents. The most recent Poisons (Amendment) Act 2022 (Act A1666) introduced administrative and enforcement updates but did not address this regulatory gap. As a result, the legal position remains unclear, and optometrists—particularly in private practice—continue to rely on ophthalmological supervision to perform cycloplegic refraction. This ambiguity in scope and regulation limits the broader adoption of cycloplegia in paediatric refractive assessment, especially in community-based settings [8, 9].

Although basic refractive services are available through the public sector in Malaysia, spectacles are mainly provided by the private sector. Spectacles are regarded as non-medical optical devices and are not included in national health financing schemes [10, 11]. Public sector facilities usually do not provide optical dispensing services, and cycloplegia may not be available at many private optical outlets [9, 12]. These systemic gaps often require families to visit multiple locations for eye examinations, refraction, and spectacle acquisition, potentially delaying care and limiting access—particularly for younger children who require cycloplegic refraction [9, 13, 14].

This study aims to describe paediatric refraction practices in Kuala Lumpur, with a focus on the use of cycloplegic agents. It examines differences across professional groups and sectors (public vs. private) and identifies barriers to refraction and cycloplegia in children. Given its exploratory nature, a descriptive cross-sectional survey with supplementary analyses was conducted to summarise practice patterns and trends.

## Methods

This study reports quantitative findings from a two-month (June–July 2018) mixed-methods investigation of paediatric refraction practices among eye care professionals in Kuala Lumpur. A three-stage recruitment approach was used to identify suitable participants and improve response rates through both digital and face-to-face methods.

### Inclusion and exclusion criteria

Eligible participants were licensed ophthalmologists, optometrists, and opticians practising within 50 km of Kuala Lumpur city centre, chosen to reflect metropolitan eye care delivery. Professionals who did not provide informed consent were excluded from participation.

### Data collection

Stage 1 involved identifying potential participants through Malaysia’s professional and regulatory bodies. Contact details were obtained via professional listings, email outreach, and direct searches. Personalised invitations were emailed and sent via WhatsApp to license eye care professionals.

In Stage 2, an online survey was created using Google Forms and shared through professional networks, WhatsApp, and Facebook. Despite wide distribution, the response rate was low (1–3%), with 21 replies from 494 emails and 158 WhatsApp messages.

Stage 3 involved purposive field visits using a random-walk method to supplement participation and reach professionals identified in Stage 1. These visits targeted optical premises, clinics, and hospitals across Kuala Lumpur. During these visits, most participants completed the survey on-site, which took less than 10 min. For participants identified during Stage 1, information sheets and consent forms were shared electronically in advance. For those recruited via random-walk field visits in Stage 3, documents were provided on-site prior to participation, and sufficient time was given to review before consent was obtained. Qualitative interviews, lasting around 30 min, were scheduled separately and are not included in this manuscript.

### Survey instrument

A multidisciplinary team with expertise in optometry, public health, and law developed the survey. Because no standard instrument was available, the questionnaire was developed from a literature review and consultations with MSc Public Health Eye Care professionals. The draft instrument was designed by EBH and reviewed for content validity by PM. It was piloted among MSc PHEC eye care professional students at the London School of Hygiene and Tropical Medicine. Following the pilot, no amendments were necessary, as the survey was clear, relevant, and easy to complete.

The final questionnaire consisted of twenty items covering: (1) Demographics and qualifications (e.g., years of experience, professional background). (2) Clinical practice (e.g., number and age distribution of children examined monthly). (3) Use of cycloplegia (e.g., frequency and age group breakdown). (4) Reasons for non-use (e.g., barriers to refraction or cycloplegia).

Broad definitions were used to account for variation in local paediatric refraction practices: “examination” referred to any form of visual assessment; “refraction” to the objective or subjective measurement of refractive power; “cycloplegia” to the diagnostic use of cycloplegic agents. Survey responses were checked for completeness. Qualitative interviews with a subset of participants were conducted separately and are omitted here.

### Sample size consideration

A non-probability purposive sample was used to capture a cross-section of paediatric refraction practices in Kuala Lumpur. No formal sample size calculation was performed; the goal was to maximise participation across professional groups. Out of 726 professionals contacted, 222 completed the survey. This approach aligns with the study’s exploratory purpose and the practical challenges of engaging clinical professionals in voluntary survey research.

### Statistical analysis

The data were anonymised and de-identified before analysis. Microsoft Excel (Microsoft 365 MSO, Version 2309, Build 16.0.1.16827.20278, 64-bit) was used for data cleaning. All statistical analyses were conducted using a reproducible Python-based pipeline (Python version 3.12.3; pandas 2.2.2; SciPy), including descriptive statistics and exploratory inferential analyses. The primary aim was to provide an overview of paediatric refraction practices among eye care professionals in Kuala Lumpur. Given the exploratory nature of this study and the purposive, non-probability sampling design, descriptive statistics were considered most appropriate to summarise observed practice patterns. Only complete surveys were analysed.

Frequency counts and percentages were calculated by sector (public vs. private) and professional group (ophthalmologists, optometrists, opticians). Tables display respondent demographics and the number of professionals who examine, refract, and use cycloplegia across four age groups (< 4 years, 5–9 years, 10–14 years, 15–18 years), reflecting emmetropisation and school-age myopia progression [1, 3]. Graphs illustrate the proportion of professionals performing examinations, refractions, and cycloplegia across these age groups.

Exploratory inferential analyses were conducted to illustrate sectoral differences. Chi-square tests of independence compared cycloplegia use between public and private optometrists across age groups. Odds Ratios (ORs) with 95% confidence intervals were calculated post hoc for the most clinically relevant comparison (public vs. private optometrists, children aged 5–9 years). Spearman’s rank correlation assessed age-related trends in cycloplegia use across sectors. These inferential results are reported in Supplementary File 1 and are presented as exploratory, not generalisable to the broader population.

## Results

Two hundred and twenty-two eye care professionals completed the survey during the study period (June–July 2018), representing a 30.5% response rate from the 726 individuals contacted via email or WhatsApp. The survey collected information on respondents’ demographics, work settings, professional qualifications, and experience in conducting paediatric eye examinations. As shown in Table 1, most respondents worked in the private sector (61.3%), with a male-to-female ratio of 1:2. Many participants were of Malay (54%) or Chinese (42%) ethnic background. The majority held a bachelor’s degree and were optometrists (65%). Some respondents were opticians with either a high school certificate (4%) or a diploma (17%), whilst a minority were ophthalmologists with a master’s degree (14%). Additionally, about three-quarters of the respondents had six or more years of experience conducting eye examinations on children.

**Table 1:** Respondent demographics, including work setting, gender, ethnicity, academic qualifications for examining children, and years of experience in examining children

	*n* (%) of respondents Total: 222
Work setting	
Public Sector	86 (39)
Private Sector	136 (61)
Gender	
Female	144 (65)
Male	78 (35)
Ethnicity	
Malay	118 (54)
Chinese	94 (42)
Indian	8 (4)
Other	2 (1)
Academic qualification for examining children	
High school certificate (Optician)	9 (4)
Diploma (Optician)	38 (17)
Bachelor’s degree (Optometrist)	145 (65)
Master’s degree (Ophthalmologist)	30 (14)
Years of performing eye examinations for children	
< 2 years	13 (6)
2–5 years	44 (20)
6–10 years	64 (29)
> 10 years	101 (45)
Mean	11.36 ± 8.12 (SD)

### Paediatric refraction practice based on the working sector

**Fig. 1:**
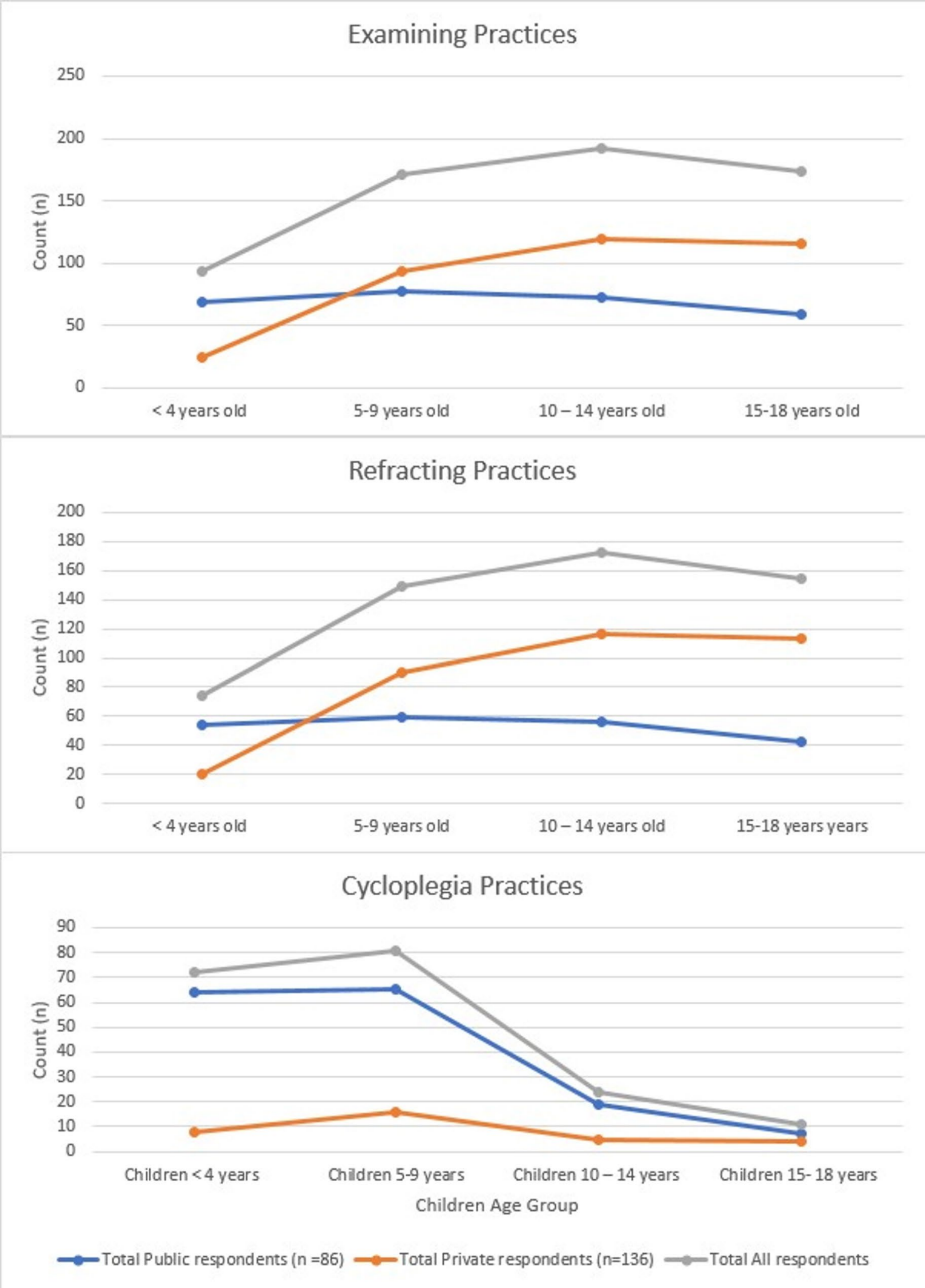
Trends in paediatric refraction practice for examining, refracting, and performing cycloplegia on children across age groups based on respondents from public, private, and all sectors

Among the 222 respondents, most (61.3%) worked in the private sector, focusing mainly on children aged 10–14 years, while public sector professionals (38.7%) focused more on children aged 5–9 years. Cycloplegia was most used by public-sector respondents for children aged 5–9 years (75.6%), but it was rarely employed in the private sector across all ages (Table 2). Refraction rates and eye examinations were highest among children aged 10–14 years, although cycloplegia use declined among older children across all sectors (Fig. 1). Exploratory inferential results comparing sectoral differences are presented in Supplementary File 1.

**Table 2:** Count (*n*) and percentage (%) of ophthalmologists, optometrists, and opticians from public and private working sectors examining, refracting, and cycloplegia children across age groups

	Examining, *n* (%)				Refracting, *n* (%)				Cycloplegia, *n* (%)			
	Age < 4 years	Age 5 – 9 years	Age 10 – 14 years	Age 15- 18 years	Age < 4 years	Age 5 – 9 years	Age 10 – 14 years	Age 15- 18 years	Age < 4 years	Age 5 – 9 years	Age 10 – 14 years	Age 15- 18 years
Public Sector												
Ophthalmologist (*n*=24)	17 (70.8)	20 (83.3)	19 (79.2)	18 (75)	2 (8.3)	3 (12.5)	3 (12.5)	2 (8.3)	13 (54.2)	14(58.3)	3 (12.5)	1 (4.2)
Optometrist (*n* =62)	52 (83.9)	57 (91.9)	54 (87.1)	41 (66.1)	52(83.9)	56 (90.3)	53 (85.5)	40 (64.5)	51 (82.3)	51(82.3)	16(25.8)	6 (9.7)
Total Public (*n* =86)	69 (80.2)	77 (89.5	73 (84.9)	59 (68.6)	54(62.8)	59 (68.6)	56 (65.1)	42 (48.8)	64 (74.4)	65(75.6)	19(22.1)	7 (8.1)
Private Sector												
Ophthalmologist (*n*=6)	5 (83.3)	6 (100.0)	6 (100.0)	6 (100.0)	2 (33.3)	3 (50.0)	3 (50.0)	3 (50.0)	2 (33.3)	3 (50.0)	0 (0.0)	1 (16.7)
Optometrist (*n*=83)	15 (18.1)	61 (73.5)	70 (84.3)	67 (80.7)	14(16.9)	60 (72.3)	70 (84.3)	67 (80.7)	6 (7.2)	13(15.7)	5 (6.0)	3 (3.6)
Optician (*n*=47)	4 (8.5)	27 (57.4)	43 (91.5)	42 (89.4)	4 (8.5)	27 (57.4)	43 (91.5)	43 (91.5)	0 (0.0)	0 (0.0)	0 (0.0)	0 (0.0)
Total Private (*n*=136)	24 (17.6)	94 (69.1)	119(87.5)	115 (84.6)	20 (14.7)	90 (66.2)	116 (85.3)	113(83.1)	8 (5.9)	16 (11.8)	5 (3.7)	4 (2.9)
Total Respondents (*n*=222)	93 (41.9)	171 (77.0)	192 (86.5)	174 (78.4)	74 (33.3)	149 (67.1)	172 (77.5)	155 (69.8)	72 (32.4)	81 (36.5)	24 (10.8)	11 (5.0)

### Paediatric refraction practice based on a professional group

**Fig. 2:**
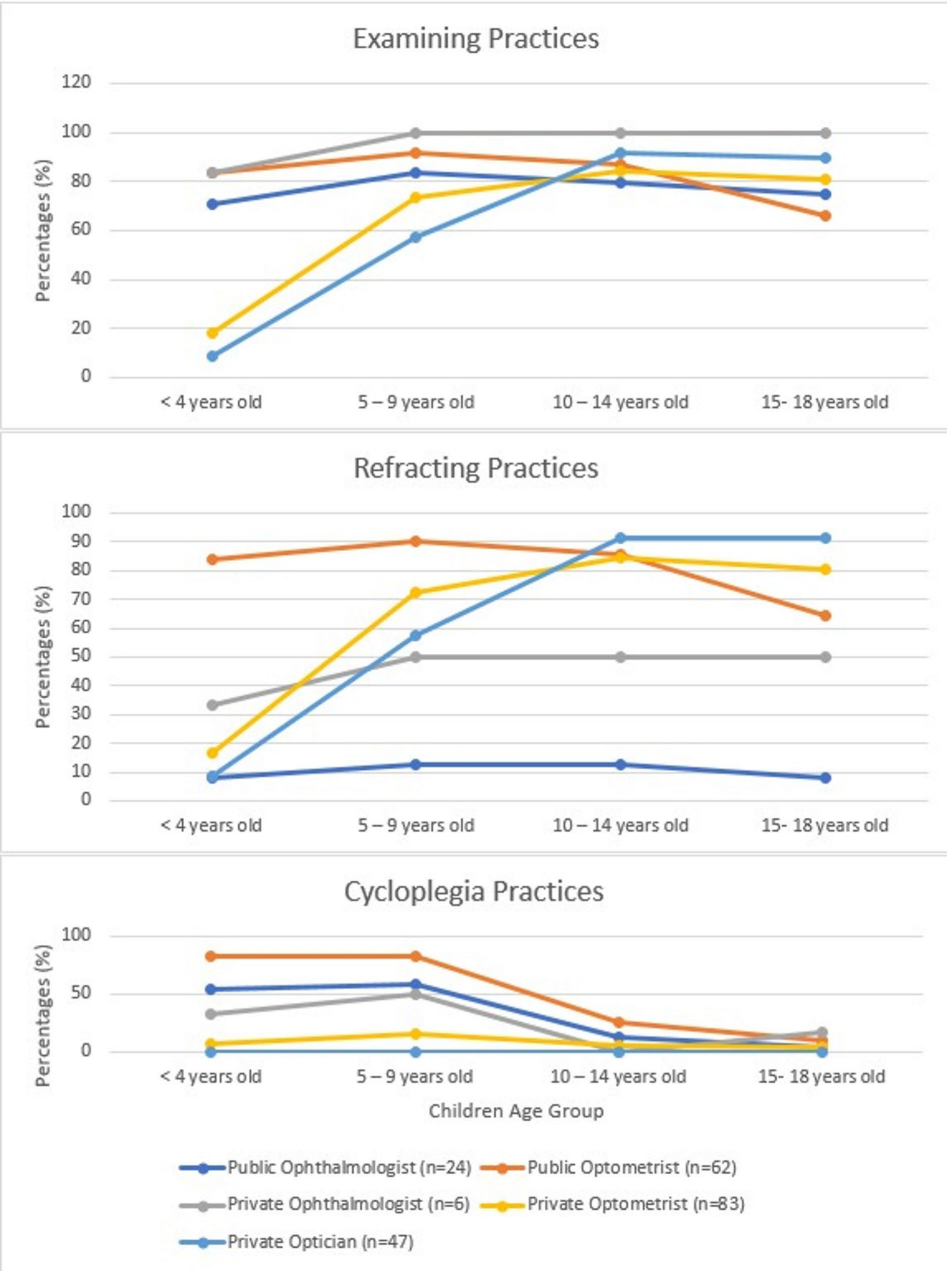
Paediatric refraction practice of respondents, based on professional qualifications and working sectors, in examining, refracting, and performing cycloplegia on children across various age groups

Patterns varied by profession (Table 2; Fig. 2, Supplementary Table S1). Cycloplegia use was common among public optometrists in younger children: 82.3% in those < 4 years, compared with 7.2% in private practice (χ² = 83.74, df = 1, *p* < 0.001). In the 5–9-year group, 82.3% of public optometrists used cycloplegia, compared with only 15.7% in private practice. This difference corresponded to an Odds Ratio of 24.97 (95% CI 10.35–60.21; χ² = 63.84, df = 1, *p* < 0.001). Cycloplegia use declined in older children (15–18 years): 9.7% in the public sector and 3.6% in private practice. The difference was not statistically significant (Fisher’s Exact Test, OR = 2.86, 95% CI 0.69–11.91, *p* = 0.1715).

Ophthalmologists, especially in the public sector, rarely performed refractions themselves (only 8.3% in the 5–9-year group), reflecting task delegation within hospitals. Opticians, exclusively in the private sector, reported 0% cycloplegia use across all age groups, consistent with scope-of-practice restrictions.

### Practice variation and statistical differences

All inferential analyses were conducted using a reproducible Python-based pipeline (see Supplementary File 1). Supplementary File 1 provides the complete exploratory inferential analyses, including chi-square and Fisher’s Exact tests, quantifying sectoral and age-related differences in cycloplegia use among optometrists. These results support the descriptive patterns reported above: significant disparities in younger age groups (< 4 and 5–9 years), a small but still statistically significant difference at 10–14 years, and no significant difference at 15–18 years.

Trend analysis demonstrated a decreasing pattern of cycloplegia use with increasing age among public-sector optometrists, while usage remained consistently low across all age groups in the private sector. Given the limited number of age categories, these findings are interpreted as descriptive patterns rather than definitive inferential results.

Figures 1 and 2 illustrate these trends visually, highlighting contrasts between public- and private-sector practice.

### Barriers to refracting and using cycloplegia

Table 3 summarises the proportion of professionals who reported not performing paediatric refraction or not using cycloplegia across professional groups. The distribution of reported reasons for these behaviours is presented in Supplementary Table S2, with additional free-text responses detailed in Supplementary File 2- Appendix 2.

**Table 3:** Paediatric refraction and cycloplegia patterns among eye care professionals in Kuala Lumpur (*n* = 222)

Professional group	*n*	Not refracting *n* (%)	Never use cycloplegia *n* (%)
Public Ophthalmologist	24	23 (95.8%)	9 (37.5%)
Public Optometrist	62	6 (9.7%)	2 (3.2%)
Private Ophthalmologist	6	3 (50.0%)	1 (16.7%)
Private Optometrist	83	11 (13.3%)	66 (79.5%)
Private Optician	47	3 (6.4%)	47 (100.0%)

Percentages are calculated within each professional group. “Not refracting” refers to respondents with a non-zero response for “reason for not refract” (norefreas ≠ 0). “Never use cycloplegia” refers to respondents reporting no cycloplegia use (oftencyclo = 0), excluding missing values. Variable definitions are provided in Supplementary File 2

Among public-sector ophthalmologists, the most common reason for not refracting children was *‘Not my job/work scope’*. In contrast, among private-sector optometrists who never used cycloplegia, the most frequently reported reason was that it was *‘Prohibited by law’* (79.5%). Public-sector respondents more commonly selected ‘Others’, reflecting routine access to diagnostic drugs within institutional settings.

Supplementary Table S3 further demonstrates differences in access to cycloplegic agents. Public-sector professionals predominantly obtained cycloplegic drugs through institutional sources such as employers or hospital pharmacies, whereas a large proportion of private-sector optometrists and opticians reported *“Not applicable”*, suggesting limited access or regulatory constraints.

**Fig. 3:**
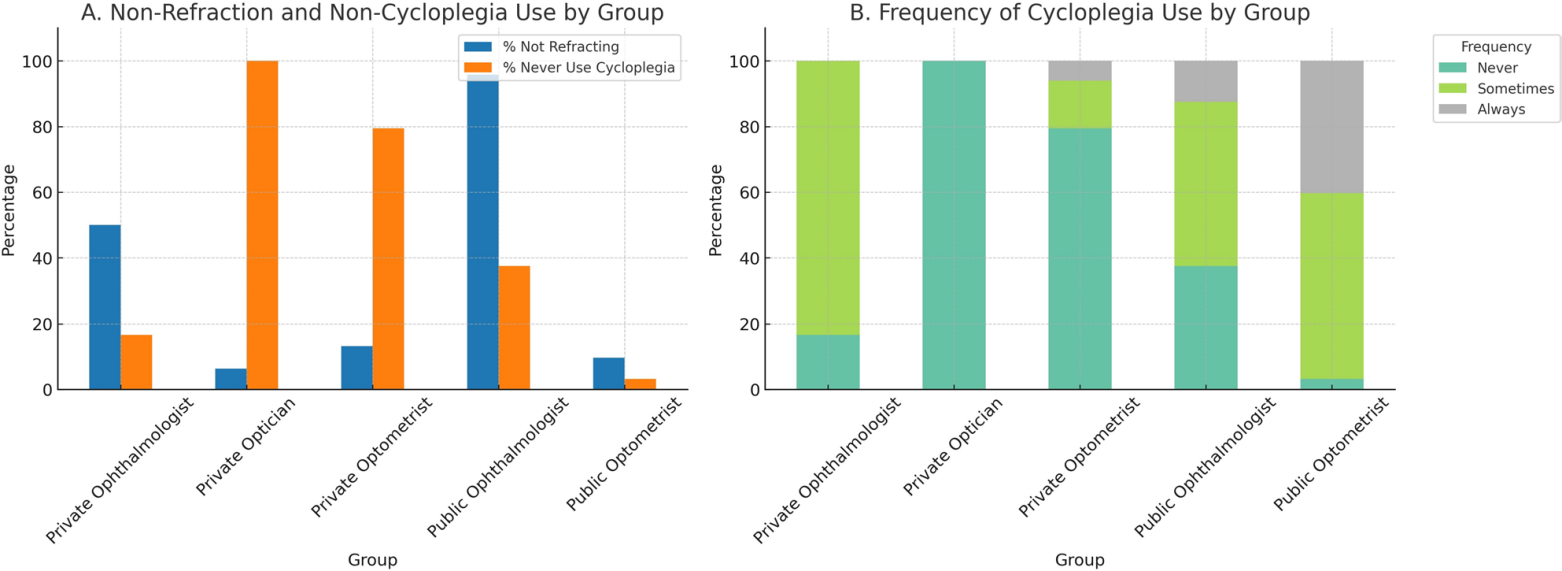
Paediatric refraction and cycloplegia practices among professional groups. Note: Panel **A** shows the percentage of respondents in each professional group who reported never refracting children and never using cycloplegia. Public ophthalmologists reported the highest rates of non-refraction, while private optometrists and opticians showed the highest rates of cycloplegia non-use. Panel **B** displays the frequency of cycloplegia use, showing that public-sector professionals were more likely to report “always” or “sometimes” using cycloplegia. In contrast, private sector professionals were more likely to report “never” use. These patterns may reflect institutional, regulatory, and perceptual differences across sectors

Supplementary File 2 also details free-text responses, highlighting additional constraints such as limited drug availability, restrictive organisational policies, perceived regulatory limitations, and uncertainty regarding the clinical necessity of cycloplegia in paediatric examinations. These patterns are illustrated in Fig. 3 (Panels A and B), which summarises differences in refraction practices and cycloplegia use across professional groups. Detailed distributions of reported barriers are presented separately in Supplementary Table S2 and Supplementary File 2- Appendix 2.

### Summary of trends

Across all analyses, a consistent pattern emerged: public-sector optometrists provided more comprehensive paediatric refractive care—including routine cycloplegia—particularly for younger children. Private-sector optometrists and opticians tended to examine older children and reported limited access, authority, or perceived legal permission to use diagnostic agents.

These overall trends align with the descriptive results in Table 2; Figs. 1 and 2, as well as the exploratory statistical findings presented in Supplementary File 1, and the distribution of reported barriers in Supplementary Table S2.

In summary, public-sector optometrists delivered the most comprehensive paediatric refractive services, while private-sector practitioners reported limited cycloplegia use, reflecting differences in scope, access, and regulation.

## Discussion

This study is the first in Kuala Lumpur (KL), Malaysia, and the Western Pacific region to examine how different eye care professionals perform refraction in children. Clear contrasts emerged: public-sector professionals were more likely to examine younger children and to employ cycloplegia, whereas private-sector practitioners focused on older children and often refracted without cycloplegia.

A comparable UK survey reported similar trends: 66% of optometrists did not assess children under 2 years, and one-third routinely performed cycloplegia in 2–4-year-olds, closely aligning with our KL findings [15]. In both settings, the use of cycloplegia decreased markedly among older children. Substantial variation was observed among professional groups in KL.

These variations reflect a combination of systemic and perceptual barriers. Public-sector ophthalmologists most frequently reported not performing paediatric refraction, citing role delegation as the primary reason. Private-sector optometrists demonstrated the highest proportion of non-cycloplegic practice, often citing legal or regulatory constraints, while opticians reported limitations related to scope-of-practice. These findings are consistent with prior Malaysian studies reporting private optometrists’ focus on older children and limited use of retinoscopy, as well as qualitative reports of time constraints [16, 17]. Although time is frequently cited in the literature [14], only 1.4% of respondents in this study identified it as a barrier.

The persistence of regulatory ambiguity exacerbates these challenges. The Optical Act 1991 provides a framework for vision examinations involving diagnostic agents, but the Poison Act 2022 does not explicitly permit optometrists to store cycloplegic agents independently [7, 10]. Consequently, some private-sector optometrists remain dependent on ophthalmological supervision or facilities with limited access to diagnostic drugs. These structural differences in access are further illustrated in Supplementary Table S3, which demonstrates the disparity in cycloplegic drug availability between public and private sector practitioners. This results in a fragmented care pathway: public-sector professionals who perform cycloplegia often do not dispense spectacles, while private-sector practitioners with dispensing capabilities frequently lack access to diagnostic agents. Families may therefore require multiple visits across sectors to obtain a complete eye examination and prescription, contributing to delays and inequities in care [8, 18].

It is also important to recognise that legal or access barriers do not solely determine cycloplegia use. Clinical discretion plays a significant role, and clinicians may choose not to use cycloplegia based on factors such as the child’s age, level of cooperation, presenting symptoms, or anticipated need for further examination [1, 3] Practice protocols and workflow constraints may further influence decision-making. These considerations emphasise that cycloplegia use is not strictly binary but context dependent. Cycloplegia should be employed when it enhances diagnostic accuracy or patient safety, while omission may be appropriate when clinical circumstances do not warrant its use. This underscores the importance of balancing regulatory clarity with clinical judgment in paediatric eye care.

Integrating optometry services into ophthalmology departments can promote a more structured and efficient paediatric refraction workflow within the public sector [8, 9, 12]. Task delegation allows optometrists to perform cycloplegic refraction while ophthalmologists focus on ocular disease management, thereby optimising patient flow in high-volume settings. However, refractive care remains incomplete without access to spectacle dispensing within public facilities. Policymakers should consider incorporating basic dispensing services or establishing coordinated referral pathways between public and private providers to improve continuity of care [11, 19].

Recent policy developments indicate progress toward reform. In 2025, the Malaysian Optical Council (MOC) introduced the *Garis Panduan Penggunaan Ubat Diagnostik yang Mengandungi Racun bagi Perkhidmatan Optometris Swasta* (Guideline on the Use of Diagnostic Drugs Containing Poisons for Private Optometric Practice), endorsed by the Poisons Board at its 99th meeting [20]. The guideline authorises registered optometrists who have completed the Enhanced Eye Health Care (EEHC) module to use diagnostic agents— including *cyclopentolate*,* tropicamide*,* and proparacaine*—for cycloplegic refraction and pupil dilation. While it does not confer new statutory rights, it represents the first formal administrative recognition of optometrists’ ability to handle controlled diagnostic drugs independently under defined conditions.

This development reflects broader regional trends. In Singapore, Yap and Mishu (2024) reported similar discussions on expanding optometric prescribing privileges to improve access to paediatric myopia management [21]. Collectively, these development suggest a gradual alignment of Malaysian optometric practice with international competency standards and may support future legislative reform. In the interim, the 2025 MOC guideline has the potential to enhance professional standardisation and patient safety, encouraging more consistent cycloplegic refraction practices nationwide.

Differences in clinical practice between sectors continue to influence access to comprehensive paediatric refractive services. Strengthening training, improving resource allocation, and promoting inter-professional collaboration may help ensure that children receive appropriate and timely care [8]. Shared-care models—where optometrists are formally integrated into ophthalmology services to perform cycloplegic refraction—may further optimise service delivery, as suggested by our findings [9]. Future research should explore strategies to improve accessibility and efficiency of cycloplegic refraction across both public and private eye care systems [19].

Cycloplegic refraction remains essential not only for detecting latent hyperopia, amblyopia, and accommodative esotropia, but also for supporting accurate myopia management—a growing public health concern associated with sight-threatening complications such as retinal detachment and glaucoma [4, 5, 22].

In summary, differences in scope, regulatory frameworks, and access to diagnostic agents continue to shape paediatric refractive care in Malaysia. Addressing these factors through coordinated policy reform and integrated care models will be essential to ensure equitable and effective services for children.

### Strengths and limitations

This mixed-method study design effectively explored paediatric refraction practices across diverse professional groups within a relatively short period. The purposive sampling, combined with a random-walk recruitment strategy, facilitated face-to-face engagement with participants, improving response rates and enabling in-depth contextual understanding. Although the qualitative component is not reported here, it complemented the quantitative findings by informing questionnaire design and interpretation. The novelty of this approach lies in its focus on a niche yet important area of clinical practice among eye care professionals from both public and private sectors in Kuala Lumpur, which adds significant strength to the study.

However, several limitations should be acknowledged. The study employed a non-representative, convenience sampling approach. It achieved a modest response rate, which limits the generalisability of the findings to the broader population of Malaysian eye care professionals. The purposive design, while useful for exploratory research, inherently restricts the ability to draw inferential statistical conclusions.

Data collection took place over two months and relied on participants’ recall rather than medical records, potentially introducing reporting bias. Time and resource constraints prevented an extended data-collection phase that could have yielded more comprehensive insights. Self-reported responses might also have overestimated adherence to best paediatric refraction practices, offering a cross-sectional snapshot that may not fully reflect ongoing trends.

Future research should consider collaborating with professional organisations to boost response rates and adopting more rigorous probability-based sampling methods. Extending the study’s duration and including perspectives from parents, guardians, and children across different regions of Malaysia—including both urban and rural areas—would yield a more comprehensive understanding of paediatric refraction practices and access barriers.

## Conclusion

Building on the Discussion, this study underscores how sectoral differences, regulatory ambiguity, and clinical discretion collectively shape paediatric refractive care in Kuala Lumpur. These findings highlight both systemic barriers and emerging policy solutions, framing the need for reforms that balance regulatory clarity with respect for professional judgment.

This study highlights substantial variation in paediatric refraction practices across Kuala Lumpur, shaped by children’s age, professional roles, and employment sector. Private-sector practitioners reported the most significant challenges in accessing cycloplegic agents—critical for accurate paediatric refractive assessment—due to regulatory ambiguity, perceived legal restrictions, and limited institutional support. These barriers contribute to uneven service delivery and reduced uptake of clinically recommended cycloplegia in community settings.

Improving access to cycloplegic drugs for qualified practitioners, especially in private practice, is essential for ensuring accurate prescriptions, detecting latent hyperopia and accommodative disorders, and preventing long-term visual impairment in children. Strengthening clarity around professional scope and guaranteeing consistent availability of diagnostic agents would help standardise care across sectors.

The recently introduced 2025 Malaysian Optical Council Guideline, which permits trained optometrists to use specific diagnostic agents under defined conditions, represents a timely and essential step toward addressing these gaps. While it does not resolve all regulatory uncertainties, it provides the first structured administrative pathway for safer, more consistent cycloplegic practice nationwide. Continued policy development, coupled with workforce training and aligned service models, will be key to advancing equitable paediatric eye care in Malaysia.

Looking ahead, aligning regulatory frameworks with clinical discretion and expanding shared care models will be critical to ensuring that paediatric refractive services in Malaysia not only meet international competency standards but also deliver equitable, accessible care for all children.

## Supplementary Information

Supplementary Material 1.

Supplementary Material 2.

Supplementary Material 3.

## Data Availability

This published article and its supplementary files include all data generated or analysed during this study. Additional anonymised datasets are available from the corresponding author upon reasonable request for non-commercial academic use—supplementary materials provided coded variables and summary tables in machine-readable format to support transparency and reuse.
