# Supplementary material for: Paediatric refraction practices: access to cyclopegia, barriers, and disparities among eye care professionals in Kuala Lumpur
Source: BMC Pediatr. 2026 Jun 5;26:736. doi: 10.1186/s12887-026-07069-x (PMC13471644; doi:10.1186/s12887-026-07069-x)
Supplement: Supplementary file 1 [file 12887_2026_7069_MOESM1_ESM.pdf]

KUALA LUMPUR CHILDREN REFRACTION SURVEY

~note : children in this study context are person below the age of 18 years old~

1. Subject ID: ☐☐☐

2. Setting: Public Sector O (1) *(mark)*
Private Sector O (2)

3. Participant has read and understood the Information Sheet: YES O (1) NO O (2)

4. Interviewer has received the signed consent form: YES O (1) NO O (2)

Subject Socio-Demographic Details

5. Sex : Male O (1)
Female O (2)

6. Ethnicity Malay O (1)
Chinese O (2)
Indian O (3)
Bumiputra O (4)
Other O (5)
Specify if other ______

7. Nationality: Malaysian citizen O (1)
Malaysian PR O (2)
Foreign citizen O (3)
Other O (4)
Specify if other ______

Subject Qualification & Experience Status

8. What is the highest ACADEMIC qualification that you have that allow you to conduct eye examinations for children?
High School Certificate O (1)
Diploma O (2)
Bachelor Degree O (3)
Master Degree O (4)
Others O (5)
Specify if other ______

9. When do you start conducting eye examination for children?
In year ☐☐☐☐

Children as Patients

10. How many children (defined as under 18) did you examined in the past 30 days? ☐☐☐ children *(0 if none)*

11. Among these, how many belonged to each of the following age groups?
less than 4 years? ☐☐☐ *(0 if none)*
between 5 to 9 years? ☐☐☐ *(0 if none)*
between 10 to 14 years? ☐☐☐ *(0 if none)*
between 15 to 18 years? ☐☐☐ *(0 if none)*

12. Did you performed any refraction for children in the past 30 days?
YES O (1) *If YES skip to question 14*
NO O (2) *If NO answer question 13* and skip question 14-15

13. What is the main reason for not performing any refraction for children in the last 30 days?
no my job/work scope O (1)
perform refraction on adults only (above 18 years) O (2)
too busy O (3)
too complicated O (4)
Others O (5)
Specify if *'Others'* ______

Refraction For Children

14. Among the [x] number of children you examined in the past 30 days, how many did you refract?

☐☐☐ x = Answer given to question 10

(*0 if none*)

15. Among these, how many belonged to each of the following age groups?

less than 4 years?	☐☐☐	(*0 if none*)
between 5 to 9 years?	☐☐☐	(*0 if none*)
between 10 to 14 years?	☐☐☐	(*0 if none*)
between 15 to 18 years?	☐☐☐	(*0 if none*)

16. How often do you use cycloplegic drugs on children during refraction?

O (a) Never *(answer Q17 skip to Q 20)*
O (b) Sometimes *(skip to Q 17)*
O (c) Always *(skip to Q 17)*

17. What is the main reason for NOT using cycloplegic drugs?

waste of time and resources O (1)
difficult and complicated O (2)
not important O (3)
Prohibited by law O (4)
Using non-drug method to aid refraction for example, fogging or 'Mohindra' technique O (5)
Painful to the eye O (6)
Parents do not give consent O (7)
Children became agitated O (8)
Specify if *'Others'* ______________________ Others O (9)

18. Among the children on whom you used cycloplegic drugs during refraction assessment in the past month, how many belonged to each of the following age group?

less than 4 years	☐☐☐	(*0 if none*)
between 5 to 9 years	☐☐☐	(*0 if none*)
between 10 to 14 years	☐☐☐	(*0 if none*)
between 15 to 18 years	☐☐☐	(*0 if none*)

19. Where do you get the supply of this drug?

supplied by my employer O (1)
buy it from supplier O (2)
buy from pharmacy O (3)
buy from other eye care practitioner O (4)
Specify if *'Others'* ______________________ Others O (5)

Opinion on Importance of Cycloplegic Drugs in Children

20. How important do you think it is to use cycloplegic drugs for refraction for children?

(a) below the age of 4 years old?
Very important O (1)
Quite important O (2)
Not important O (3)

(b) between the age of 5 to 9 years old?
Very important O (1)
Quite important O (2)
Not important O (3)

(c) between the age of 10 to 14 years old?
Very important O (1)
Quite important O (2)
Not important O (3)

(d) between the age of 15 to 18 years old?
Very important O (1)
Quite important O (2)
Not important O (3)
