# Supplementary material for: Paediatric refraction practices: access to cyclopegia, barriers, and disparities among eye care professionals in Kuala Lumpur
Source: BMC Pediatr. 2026 Jun 5;26:736. doi: 10.1186/s12887-026-07069-x (PMC13471644; doi:10.1186/s12887-026-07069-x)
Supplement: Supplementary file 2 [file 12887_2026_7069_MOESM2_ESM.docx]

**Comparative Analysis of Cycloplegia Use by Public vs Private Sector Optometrists Across Child Age Groups**

**Objective:**
To examine sectoral differences in cycloplegia use among optometrists across paediatric age groups, we performed exploratory comparative analyses between public and private sectors.

**Rationale:**
These analyses were conducted within a single professional group (optometrists) and are presented as supplementary evidence to support descriptive trends observed in the main manuscript. Given the non-probability sampling design, findings are not intended for population-level inference.

**Table S1. Comparison of cycloplegia use between public and private sector optometrists across age groups**

| Age group | Test used | Statistic | p-value | OR (95% CI) |
| --- | --- | --- | --- | --- |
| <4 years | Chi-square | χ² = 83.74, df = 1 | p < 0.001 | 59.50 (20.70–171.02) |
| 5–9 years | Chi-square | χ² = 63.84, df = 1 | p < 0.001 | 24.97 (10.35–60.21) |
| 10–14 years | Chi-square | χ² = 11.21, df = 1 | p = 0.0008 | 5.43 (1.86–15.79) |
| 15–18 years | Fisher’s Exact | — | p = 0.1715 | 2.86 (0.69–11.91) |

**Note:**

Chi-square tests were used to compare cycloplegia use between public and private sector optometrists across age groups. Fisher’s Exact Test was applied where expected cell counts were less than 5. Odds ratios (ORs) with 95% confidence intervals were calculated to quantify effect sizes. Counts are based on full subgroup denominators (public n = 62; private n = 83). Analyses were performed using Python (version 3.12.3) with SciPy. These analyses are exploratory and not generalizable beyond the study sample.

**Interpretation:**

- Statistically significant differences were observed in cycloplegia use between public and private sector optometrists for children aged <4, 5–9, and 10–14 years.
- No statistically significant difference was observed for the 15–18 years age group.
- Across all age groups, cycloplegia use was consistently higher among public-sector optometrists, particularly in younger children.

**Trend Analysis of Cycloplegia Use by Public and Private Sector Optometrists Across Child Age Groups**

**Objective:**
To explore patterns in cycloplegia usage by optometrists across paediatric age groups in public and private sectors.

**Methodology:**
Cycloplegia usage proportions were calculated for four age groups (<4 years, 5–9 years, 10–14 years, and 15–18 years) separately for public- and private-sector optometrists. Spearman’s rank correlation was used to assess the monotonic trend in cycloplegia use with increasing age based on aggregated proportions.

**Results:**

**Public-sector optometrists:**

- Cycloplegia usage proportions by age group:
  - <4 years: 51/62 (82.3%)
  - 5–9 years: 51/62 (82.3%)
  - 10–14 years: 16/62 (25.8%)
  - 15–18 years: 6/62 (9.7%)
- Spearman’s rank correlation:
  - ρ (rho) = –0.949
  - p = 0.051

**Private-sector optometrists:**

- Cycloplegia usage proportions by age group:
  - <4 years: 6/83 (7.2%)
  - 5–9 years: 13/83 (15.7%)
  - 10–14 years: 5/83 (6.0%)
  - 15–18 years: 3/83 (3.6%)
- Spearman’s rank correlation:
  - ρ (rho) = –0.800
  - p = 0.200

**Interpretation:**

A decreasing trend in cycloplegia use with increasing age was observed in both sectors. Among public-sector optometrists, this trend was strong (ρ = –0.949) and approached statistical significance, whereas in the private sector, a moderate negative trend was observed but was not statistically significant.

Given the limited number of age categories (n = 4), these findings should be interpreted as descriptive patterns rather than definitive inferential results.

**Note:**

These trend analyses are exploratory and conducted within a single professional group. Spearman’s rank correlation was performed using aggregated proportions across age groups. Due to the small number of observations, statistical power is limited. Analyses were performed using Python (version 3.12.3) with SciPy. Results are not generalizable beyond the study sample.
