# Supplementary material for: Paediatric refraction practices: access to cyclopegia, barriers, and disparities among eye care professionals in Kuala Lumpur
Source: BMC Pediatr. 2026 Jun 5;26:736. doi: 10.1186/s12887-026-07069-x (PMC13471644; doi:10.1186/s12887-026-07069-x)
Supplement: Supplementary file 3 [file 12887_2026_7069_MOESM3_ESM.docx]

**Figure S2 illustrates the distribution of reasons for non-refraction and non-use of cycloplegia. The corresponding numerical distributions are provided in Supplementary Table S2.**

**Panel A** shows the distribution of stated reasons for not using cycloplegic agents among professionals who reported never using them. “Prohibited by law” was the most cited reason among private-sector professionals, while public professionals often indicated no barrier (“Not applicable”).

**Panel B** displays reasons for not refracting children among professionals who reported never performing pediatric refraction. “Not my job/work scope” was the dominant reason among public ophthalmologists. Responses marked as “Others” include free-text examples such as “no drug available,” “done by optometrist,” or “child contraindicated”. These highlight both systemic and perceptual barriers to optimal pediatric care.

**Table S2. Distribution of reported reasons for non-refraction and non-use of cycloplegia (%)**

| Professional group | Not my job/work scope (%) | Refraction for adults only (%) | Too busy (%) | Others (%) | Prohibited by law (%) | Other cycloplegia reasons (%) |
| --- | --- | --- | --- | --- | --- | --- |
| Public Ophthalmologist | 82.6 | 0.0 | 4.3 | 13.0 | 0.0 | 100.0 |
| Public Optometrist | 33.3 | 16.7 | 0.0 | 50.0 | 0.0 | 100.0 |
| Private Ophthalmologist | 33.3 | 0.0 | 0.0 | 66.7 | 0.0 | 100.0 |
| Private Optometrist | 45.5 | 9.1 | 0.0 | 45.5 | 63.6 | 36.4 |
| Private Optician | 33.3 | 33.3 | 0.0 | 33.3 | 59.6 | 40.4 |

Note: Percentages are calculated within the subgroup of respondents who reported not refracting children (for non-refraction reasons) or never using cycloplegia (for cycloplegia-related reasons). “Other cycloplegia reasons” include waste of time, difficulty, lack of importance, Mohindra/fogging technique, pain, consent issues, agitated child, and other responses. Categories are based on coded survey responses as defined in Supplementary Appendix 1**.**

**Table S3. Distribution of Cycloplegia Supply Sources by Professional Group.**

| Group | Employer | Not applicable | Pharmacy | Supplier | Total % |
| --- | --- | --- | --- | --- | --- |
| Private Ophthalmologist | 100 | 0 | 0 | 0 | 100 |
| Private Optician | 0 | 100 | 0 | 0 | 100 |
| Private Optometrist | 19.3 | 78.3 | 2.4 | 0 | 100 |
| Public Ophthalmologist | 70.8 | 20.8 | 4.2 | 4.2 | 100 |
| Public Optometrist | 91.9 | 3.2 | 0 | 4.8 | 100 |

Note: The table illustrates the percentage distribution of reported sources of cycloplegic drugs among various eye care professional groups. A large proportion of private optometrists and opticians reported “Not applicable,” indicating likely absence of access or regulatory authority. In contrast, public professionals showed more access via institutional channels, such as employer or pharmacy supply.

**Appendix 1: Data Dictionary for Supplementary File 2**

The following table provides definitions for the coded variables in Supplementary File 2, which summarise respondents' reasons for not refracting children or for not using cycloplegia.

| **Variable** | **Code** | **Meaning** |
| --- | --- | --- |
| norefreas | 1 | Not my job/work scope |
| norefreas | 2 | Refraction for adults only |
| norefreas | 3 | Too busy |
| norefreas | 4 | Too complicated |
| norefreas | 5 | Others |
| nocycloreas | 1 | Waste of time |
| nocycloreas | 2 | Difficult |
| nocycloreas | 3 | Not important |
| nocycloreas | 4 | Prohibited by law |
| nocycloreas | 5 | Mohindra/Fogging technique |
| nocycloreas | 6 | Pain |
| nocycloreas | 7 | Consent issues |
| nocycloreas | 8 | Agitated child |
| nocycloreas | 9 | Others |

These codes were used for the bar chart visualisations shown in Supplementary Figure 2.

**Appendix 2: Free-Text *“Others”* Responses for Non-Cycloplegia Reasons**

The following table presents free-text responses provided under the category “Others” by respondents who reported reasons for not using cycloplegic agents. Responses have been grouped thematically to enhance interpretability (n = 15).

| Thematic Category | Example Responses |
| --- | --- |
| Institutional or drug access issues | “No drug available”,  “Company didn’t provide”,  “Retail setting,  “No access” |
| Professional boundaries | “Because I am not a doctor”,  “Done by optometrist”,  “Not authorised” |
| Clinical reasoning | “Child can focus well”,  “Contraindicated due to allergy” |
| Regulatory confusion | “Optical Act restriction”,  “No training provided on this” |

**Appendix 3: Derived Variables and Analytical Definitions**

**Derivation of professional group from qualification**

In the absence of a directly recorded profession variable, professional group was derived from the reported highest qualification level. This approach reflects standard training pathways within the study setting.

The mapping was defined as follows:

| Qualification code | Qualification level | Derived professional group |
| --- | --- | --- |
| 1 | High school | Optician |
| 2 | Diploma | Optician |
| 3 | Degree | Optometrist |
| 4 | Master’s degree | Ophthalmologist |
| 5 | Other postgraduate qualification | Ophthalmologist |

This derived variable was combined with the working sector variable (public = 1, private = 2) to define five analytical groups:

- Public Ophthalmologist
- Public Optometrist
- Private Ophthalmologist
- Private Optometrist
- Private Optician

**Definition of analytical variables**

Participants were classified as not performing paediatric refraction if a non-zero response was recorded for the variable “reason for not refract” (norefreas ≠ 0).

Participants were classified as never using cycloplegia if “using cyclo” was coded as 0 (never), excluding missing or non-applicable responses.

**Reproducibility**

All data processing and analyses were performed using a reproducible Python-based analysis pipeline (Python 3.12.3, pandas 2.2.2).
